# Virulent genes related to the synthesis of community interaction factors among clinical samples of multidrug-resistant Acinetobacter baumannii in Iran

**DOI:** 10.3205/dgkh000552

**Published:** 2025-05-21

**Authors:** Leila Azimi, Hadi Hasani, Abdollah Karimi, Seyed Alireza Fahimzad, Ali Rezaei, Fatemeh Fallah, Shima Fatehi, Shahnaz Armin, Mohammadreza Sadr

**Affiliations:** 1 Pediatric Infections Research Center, Research Institute for Children’s Health, Shahid Beheshti University of Medical Sciences, Tehran, Iran; 2 Department of Nursing, School of Nursing and Midwifery, Shahroud University of Medical Sciences, Shahroud, Iran; 3 Multiple Sclerosis Research Center, Neuroscience Institute, Tehran University of Medical Sciences, Tehran, Iran; 4 Department of Pediatrics, School of Medicine, Sabzevar University of Medical Sciences, Sabzevar, Iran

**Keywords:** Acinetobacter baumannii, multiple drug resistance, virulence genes, biofilm

## Abstract

**Introduction:**

* Acinetobacter (A.) baumannii* poses a significant threat of resistance to multiple antibiotics. This study aimed to examine the prevalence of the *abaI* and *bap* genes in clinical isolates of multidrug-resistant *A. baumannii* collected from 10 cities in Iran.

**Method:**

Antibiotic susceptibility testing was performed using the Kirby–Bauer disk diffusion method, and multidrug resistance was confirmed using specific criteria. The presence of *abaI* and *bap* genes was identified through conventional PCR.

**Results:**

Of 50 samples total, 62% were from males, and 38% were from females, with most isolates originating from ICUs and obtained from the tracheobronchial tract. The abaI gene was present in 94% of samples, while the bap gene was present in 88%. Statistical analysis showed no significant differences in gene frequencies and antibiotic resistance patterns.

**Conclusions:**

The presence of *abaI* or *bap* genes was not related to antibiotic resistance. However, the frequency of these virulent genes was relatively high among multi-drug resistant *A. baumannii* samples. The differences in antibiotic resistance patterns of this bacterium show the need for future research in this field.

## Zusammenfassung

**Einleitung:**

*Acinetobacter (A.) baumannii* stellt eine erhebliche Gefahr auf Grund der Multiresistenz gegen Antibiotika dar. Ziel der Studie war es, die Prävalenz der abaI- und bap-Gene in klinischen Isolaten multiresistenter *A. baumannii* aus zehn Städten im Iran zu untersuchen.

**Methode:**

Die Antibiotika-Empfindlichkeit wurde mit dem Kirby-Bauer-Disk-Diffusionssuszeptibilitätstest ermittelt und die Multiresistenz wurde anhand spezifischer Kriterien bestätigt. Das Vorhandensein von abaI- und bap-Genen wurde durch konventionelle PCR nachgewiesen.

**Ergebnisse:**

Von den 50 Proben stammten 62% von Männern und 38% von Frauen, wobei die meisten Isolate von Intensivstationen stammten und aus dem Tracheobronchialtrakt gewonnen wurden. Das abaI-Gen war in 94% der Proben, das bap-Gen in 88% der Proben vorhanden. Die statistische Analyse ergab keine signifikanten Unterschiede in den Genhäufigkeiten und Antibiotikaresistenzmustern.

**Schlussfolgerung:**

Das Vorhandensein des abaI- oder bap-Gens stand in keinem Zusammenhang mit der Antibiotikaresistenz. Allerdings war die Häufigkeit dieser virulenten Gene in den Proben des multiresistenten A. baumannii relativ hoch. Die Unterschiede in den Antibiotikaresistenzmustern dieses Bakteriums zeigen die Notwendigkeit künftiger Forschung auf diesem Gebiet.

## Introduction

*Acinetobacter (A.) baumannii*, a Gram-negative bacterium, has emerged as a significant nosocomial pathogen, due to its alarming resistance to a wide array of antibiotics [1], [2]. Particularly prevalent in healthcare settings, this bacterium poses a substantial threat, leading to severe infections, especially in immunocompromised individuals [3]. The rising incidence of antibiotic resistance in *A. baumannii* raises urgent concerns, challenging conventional therapeutic approaches [4]. Understanding the intricate genetic mechanisms underlying antibiotic resistance in *A*. *baumannii* is essential for developing targeted interventions to counteract the spread of resistant strains [5]. Additionally, recent research has highlighted the crucial role of biofilm formation in *A. baumannii*, shedding light on a key adaptive strategy employed by the bacterium to bolster its resilience against antibiotics [6].

Biofilms, which are complex communities of microorganisms surrounded by their external matrix, have been implicated in the persistence and chronicity of *A. baumannii* infections [7]. The biofilm not only provides a protective shield against host immune responses but also serves as a robust defense mechanism against antibiotic penetration.

Biofilm formation in *A. baumannii* involves key genetic components, notably the *abaI* and *bap* genes [8]. The *abaI* gene, integral to quorum sensing, facilitates synchronized communication among bacterial cells, critically contributing to the coordinated development of biofilms [9]. Concurrently, the *bap* gene, responsible for encoding the biofilm-associated protein, serves as a fundamental contributor to the structural integrity of the biofilm matrix [10]. This protein enhances adhesion to surfaces and cells, markedly influencing the stability and resilience of the biofilm structure. The synergistic interplay between the *abaI* and *bap* genes underscores their pivotal roles in shaping the dynamic processes of *A. baumannii* biofilms [11]. A comprehensive understanding of these genetic elements is imperative for the formulation of targeted strategies aimed at disrupting biofilm formation, thereby presenting potential avenues for mitigating the pathogenicity and antibiotic resistance associated with *A. baumannii* infections.

This investigation focuses on unraveling the molecular mechanisms contributing to antibiotic resistance in *A. baumannii*, specifically emphasizing the pivotal role of *abaI* and *bap* genes in biofilm formation. A comprehensive understanding of these genetic elements is crucial for developing therapeutic strategies to disrupt *A. baumannii* biofilm formation, and as a result, addressing antibiotic resistance. 

The objective of this study is to assess the prevalence of the *abaI* and *bap* genes within multidrug-resistant *A. baumannii* samples obtained from 10 cities in Iran. This investigation is crucial for enhancing our understanding of the genetic landscape contributing to multidrug resistance in *A. baumannii*. The findings aim to contribute valuable insights to the ongoing efforts in developing targeted interventions and strategies to address the challenges posed by multidrug-resistant *A. baumannii* in diverse healthcare settings.

## Method

### Bacterial isolation and identification

A total of 50 clinical isolates of *A. baumannii* were obtained from diverse patients across 10 cities in Iran over two years. These samples were collected from various wards, including ICU, neurology, nephrology, gastrointestinal, surgery, orthopedics, internal, trauma, burn, pediatrics, CCU, NICU, OICU, and cardiac wards, within hospitals located in Tehran, Sanandaj, Esfahan, Hamedan, Tabriz, Mashhad, and Zahedan. The specimens, derived from blood, urine, CSF, bronchi, and trachea, were meticulously preserved at the Pediatric Infections Research Center, Research Institute of Children’s Health, Tehran, Iran. After extraction from a deep freezer at –80°C, identification and confirmation tests, including the assessment of bacterial colony morphology and biochemical tests such as oxidase and triple sugar iron agar (TSI), were conducted. Cultivation on blood agar and MacConkey agar (Merck Co., Germany) ensued, with an incubation period of 24 hours at 37°C. Further biochemical examinations for Gram-negative bacteria involved sugar fermentation, motility, citrate utilization, growth on TSI, and Indole, methyl red, Voges Proskauer, and citrate (IMVIC). The conclusive verification of *A. baumannii* isolates was achieved through the PCR method, employing specific primers targeting the *bla*_oxa51_ gene [12].

### Antibiotic susceptibility testing 

Following the confirmation of *A. baumannii* samples, we employed the Kirby–Bauer disk diffusion (DDM) method on Müller-Hinton agar (Merck Co., Germany) to identify multidrug-resistant (MDR) strains. Antibiotic susceptibility results were interpreted according to the Clinical and Laboratory Standards Institute (CLSI) guidelines. The susceptibility of MDR *A. baumannii *isolates to colistin was determined using the broth microdilution method, and the results were interpreted based on the European Committee on Antimicrobial Susceptibility Testing (EUCAST) breakpoints (resistant, >2 mg/l; susceptible, ≤2 mg/l) [13]. For classification as multidrug-resistant bacteria, *A. baumannii* isolates exhibiting resistance to three or more antimicrobial classes were considered [14].

The antibiotic disks (Mast Companies, UK) utilized included ampicillin-sulbactam (SAM), piperacillin/tazobactam (PTZ), ceftazidime (CAZ), cefepime (CPM), meropenem (MEM), imipenem (IPM), gentamicin (GEN), amikacin (AK), trimethoprim-sulfamethoxazole (SXT), ciprofloxacin (CIP), tobramycin (TN), and minocycline (MN).

### Identification of pathogenic genes 

Conventional PCR was employed to detect the virulence genes abaI and bap in samples of MDR *A. baumannii*. The primers utilized for gene identification are detailed in Table 1 (Tab. 1).

### Molecular analysis 

#### Extraction and PCR 

To conduct molecular identification of antibiotic resistance genes, DNA extraction was carried out using the Thermo America extraction kit following the manufacturer’s instructions. The procedure can be summarized as follows: Initially, 200 µl of the sample were combined with 400 µl of lysis solution in a 2 ml microtube and incubated at 65°C for 5 minutes. Subsequently, 600 µl of chloroform were promptly added, and the microtube was gently inverted several times (3 to 5 times) before being centrifuged at 10,000 rpm for 2 minutes. The precipitation solution of the kit was diluted using 720 µl of sterile deionized water plus 80 µl of 10X precipitation solution. Following centrifugation, the supernatant solution containing DNA was mixed with 800 µl of freshly prepared sedimentation solution, and the microtubes were incubated for 1 to 2 minutes at room temperature. The subsequent step involved centrifugation at 10,000 rpm for 2 minutes. To prevent the microtube from drying, the supernatant solution was carefully removed, and the DNA precipitate was dissolved in 100 µl of NaCl through slow vortexing. Cold ethanol (300 µl) was added to the solution, and the microtubes were placed in a freezer at –20°C for 10 minutes to precipitate the DNA. Following centrifugation at 10,000 rpm for 3 to 4 minutes, the sediments were washed with cold 70% ethanol. Finally, the DNA was dissolved in 100 µl of sterile deionized water using a gentle vortex.

Subsequently, PCR was conducted to identify carbapenemase genes in carbapenem-resistant gram-negative bacteria. The PCR materials were prepared in a 25 µl reaction with volumes and concentrations specified in Table 2 (Tab. 2) for each sample. These components were mixed in a 0.2 ml microtube. For multiple sample PCR, the volume and concentration of reagents were adjusted based on the number of reactions, mixed in a sterile microtube, and evenly distributed into 0.2 ml microtubes.

### CCCP (Carbonyl cyanide m-chlorophenyl hydrazone)

The responsiveness of the bacteria under examination to the antibiotic imipenem was assessed using the MIC method both independently and in conjunction with a non-specific drug pump inhibitor (carbonyl cyanide m-chlorophenyl hydrazine) through the micro broth dilution method. Utilizing Mueller Hinton broth (MHB), the studied bacteria were determined to be resistant to imipenem.

The identification of an active efflux pump in carbapenem-resistant strains of *Acinetobacter* was conducted through the use of CCCP. CCCP, having an inhibitory effect on all bacteria and efflux pumps, does not influence gene expression or impede expression but rather impacts the functionality of pump proteins. Initially, Muller’s Hinton broth was prepared, autoclaved, and subsequently divided into two groups of microplates.

The first group consisted of microplates containing Mueller Hinton broth culture supplemented with specific and consecutive concentrations of imipenem antibiotic. In contrast, the second group comprised microplates containing Mueller Hinton broth culture supplemented with designated and successive concentrations of imipenem antibiotic alongside CCCP.

In the subsequent phase of the investigation, the semi-quantitative expression of the efflux pump gene, recognized as one of the mechanisms of antibiotic resistance, was examined. To delve into the gene expression of the AdeB efflux pump, the procedure consisted of initial RNA extraction, followed by the elimination of any potential DNA in the sample, and concluding with the synthesis of cDNA.

### Extraction and real-time PCR

The total RNA of the specified bacteria was extracted utilizing an RNA extraction kit from Bioneer Korea, following the manufacturer's protocol. It is crucial to eliminate any genomic DNA from the RNA solution, as the presence of genomic DNA can lead to inaccurate positive responses in real-time PCR results concerning the determination of gene expression. To ensure the DNA-free nature of the RNA used for cDNA synthesis, a DNase I enzyme kit (1U/µl) from Fermentas was employed for DNA removal.

Following RNA extraction and validation of quality, including the absence of genomic DNA, cDNA synthesis was carried out using the AccuPower RocketScriptTM RT PreMix kit, a product of Bioneer, following the manufacturer’s instructions.

Subsequently, real-time PCR was performed on the samples to assess the semi-quantitative expression of the desired efflux pumps, utilizing primers detailed in Table 3 (Tab. 3) [15].

The real-time PCR conditions for the AdeB gene are outlined in Table 3 (Tab. 3), with the 16srRNA gene serving as a normalizer, as indicated. Following the instructions in Table 3 (Tab. 3) and Table 4 (Tab. 4), the machine was configured to progress through the required steps. Finally, the semi-quantitative expression of the efflux pump gene was computed using the formula. 2^–∆∆^*^Ct^*.

## Results

A total of 50 samples were examined, with 62.0% of the samples from males and 38.0% from females. The age range of patients from whom the samples were obtained varied from infants to 84-year-olds, with a mean age of 51.8±23.62. All samples were collected from various cities in Iran, with 34% originating from Tehran, 18% from Mashhad, and the remainder from Sanandaj, Esfahan, Hamedan, Tabriz, and Zahedan.

The majority of the MDR samples subjected to analysis were gathered from Intensive Care Units (ICUs) (61.5%), the emergency department (5.1%), and surgery wards (5.1%). Other samples were collected from various medical-surgical wards. Among the MDR samples that reached the analysis stage, 59.2% were obtained from the tracheobronchial tract, while 16.3% were derived from blood samples, and the remaining samples were collected from wounds, urine, cerebrospinal fluid (CSF), intravenous catheter sites, and bronchoalveolar lavage (BAL).

In the samples, the *abaI* gene was found at a frequency of 94%, while the *bap* gene was present in 88%, as shown in Table 4 (Tab. 4).

Disk diffusion test results, as presented in Table 5 (Tab. 5), indicated that all analyzed samples showed 100% resistance to Cefepime, Cefotaxime, Ceftazidime, Imipenem, Meropenem, and Piperacillin/Tazobactam. Colistin demonstrated the highest sensitivity at 80%, followed by Minocycline at 28%.

The Fisher’s test revealed no significant differences in the frequencies of the *abaI* and *bap* genes or the resistance patterns to various antibiotics among the studied samples. This implies that the distribution of these genetic elements and antibiotic resistance traits was uniform across the tested samples, indicating a consistent profile among the observed variables (Table 5 (Tab. 5)).

## Discussion

We examined 50 samples, with the majority being from male subjects (62.0% males vs. 38.0% females). This male predominance aligns with findings from previous studies [16], [17]. Most of the MDR samples were from the ICU (61.5%) and obtained from the tracheobronchial tract (59.2%), a pattern also consistent with previous studies [18], [19].

Based on our findings, there appears to be no association between the *abaI* gene and antibiotic resistance in *A. baumannii*. This aligns with the results of a study conducted in the western region of Iran, which investigated the correlation between extensively drug-resistant (XDR) and multidrug-resistant (MDR) *A. baumannii* and the *abaI* gene [20]. However, a study in China observed a correlation between the presence of the abaI gene and drug resistance in *A. baumannii* [9]. In our analysis, there was also no significant difference in the frequency of the *bap* gene and MDR. This finding contradicts results from other studies that have identified a relationship between the presence of the *bap* gene and subsequent biofilm formation and multidrug resistance [21], [22]. 

Several factors may contribute to this discrepancy between our results and those of other studies. Firstly, variations in the strains of *A. baumannii* studied could play a crucial role, as different strains may exhibit diverse genetic profiles and responses to antibiotic exposure [23]. Additionally, differences in experimental methods, such as variations in sample size, laboratory techniques, and testing conditions, might contribute to divergent outcomes. Environmental factors, geographical location, and the prevalence of specific strains in different regions could also impact the observed associations [24]. Furthermore, the dynamic nature of bacterial resistance mechanisms and the potential for genetic mutations over time may introduce variations between studies [25], [26]. An in-depth exploration of these factors is essential for a comprehensive understanding of the discrepancies and to refine interpretations of the relationship between the *bap* and *abaI* genes to multidrug resistance in* A. baumannii*. 

## Conclusions

Our findings indicate no significant correlation between the abaI gene and antibiotic resistance. The absence of a notable difference in the frequency of the bap gene and multidrug resistance contrasts with previous research. Several potential factors contributing to these disparities have been considered, including strain variations, methodological differences, regional influences, and the dynamic nature of bacterial resistance mechanisms. This underscores the complexity of interactions within *A. baumannii* and highlights the importance of context-specific factors in understanding gene-related phenomena. Future research should further explore these factors to enhance our understanding of the complex relationships between genes and antibiotic resistance in *A. baumannii*. Ultimately, our study contributes to the ongoing scientific discourse, emphasizing the need for nuanced interpretations and a holistic approach to comprehending microbial behavior and resistance mechanisms.

## Notes

### Competing interests

The authors declare that they have no competing interests.

### Ethical approval 

This study was approved by the ethics committee of Sabzevar University of Medical Sciences (code: IR.MEDSAB.REC.1401.111).

### Funding

This study was funded by the Sabzevar University of Medical Sciences (code: 401202).

### Acknowledgments

The authors would like to thank the Cellular and Molecular Research Center, Sabzevar University of Medical Sciences, Sabzevar, Iran. We also appreciate the help of the Pediatric Infections Research Center, Research Institute for Children’s Health, Shahid Beheshti University of Medical Sciences, Tehran, Iran.

### Authors’ ORCIDs

- Azimi L: https://orcid.org/0000-0002-7216-2530
- Hasani H: https://orcid.org/0000-0002-3070-3108
- Karimi A: https://orcid.org/0000-0003-4599-1496
- Fahimzad SA: https://orcid.org/0000-0001-6054-0656
- Rezaei A: https://orcid.org/0009-0004-3846-940X
- Fallah F: https://orcid.org/0000-0003-4455-1536
- Fatehi S: https://orcid.org/0009-0005-0914-6078
- Armin S: https://orcid.org/0000-0002-4993-482X
- Sadr M: https://orcid.org/0000-0001-5376-0933

## Figures and Tables

**Table 1:**
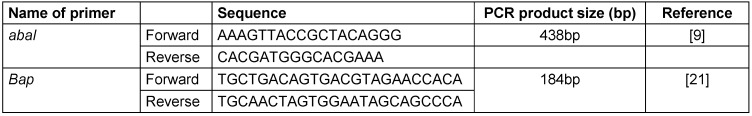
Primers used for detecting virulence-associated genes in MDR *A. baumannii* isolates

**Table 2:**
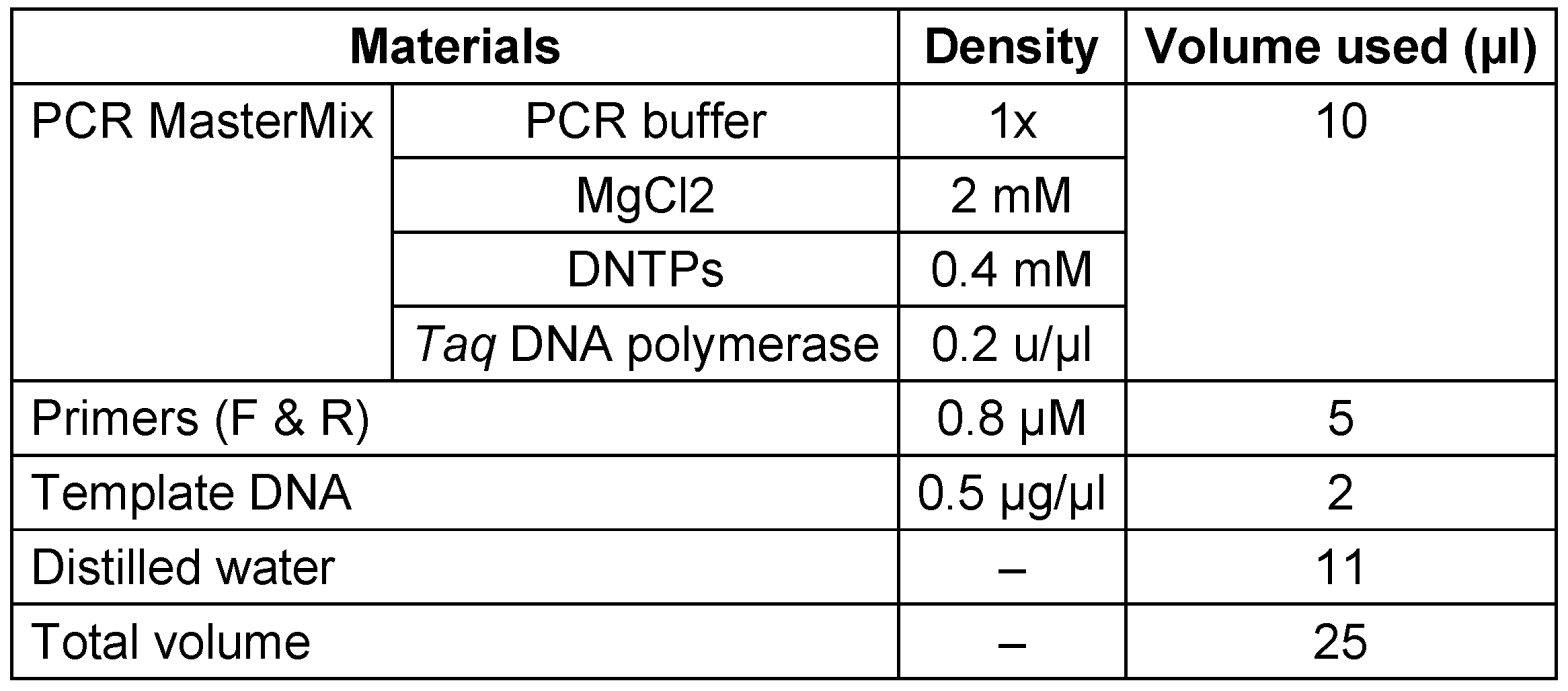
The materials used in PCR and their preparation method

**Table 3:**
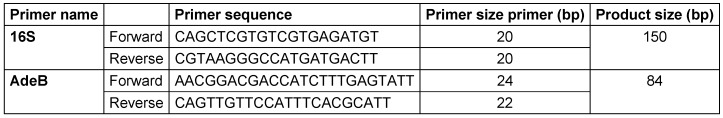
Real-Time PCR (AdeB) primers

**Table 4:**
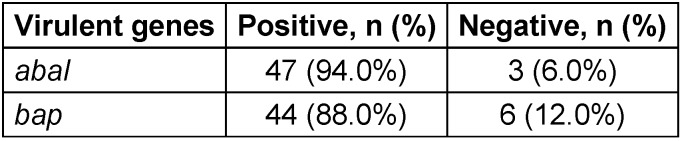
The frequency of genes related to biofilm formation in MDR *A. baumannii* samples

**Table 5:**
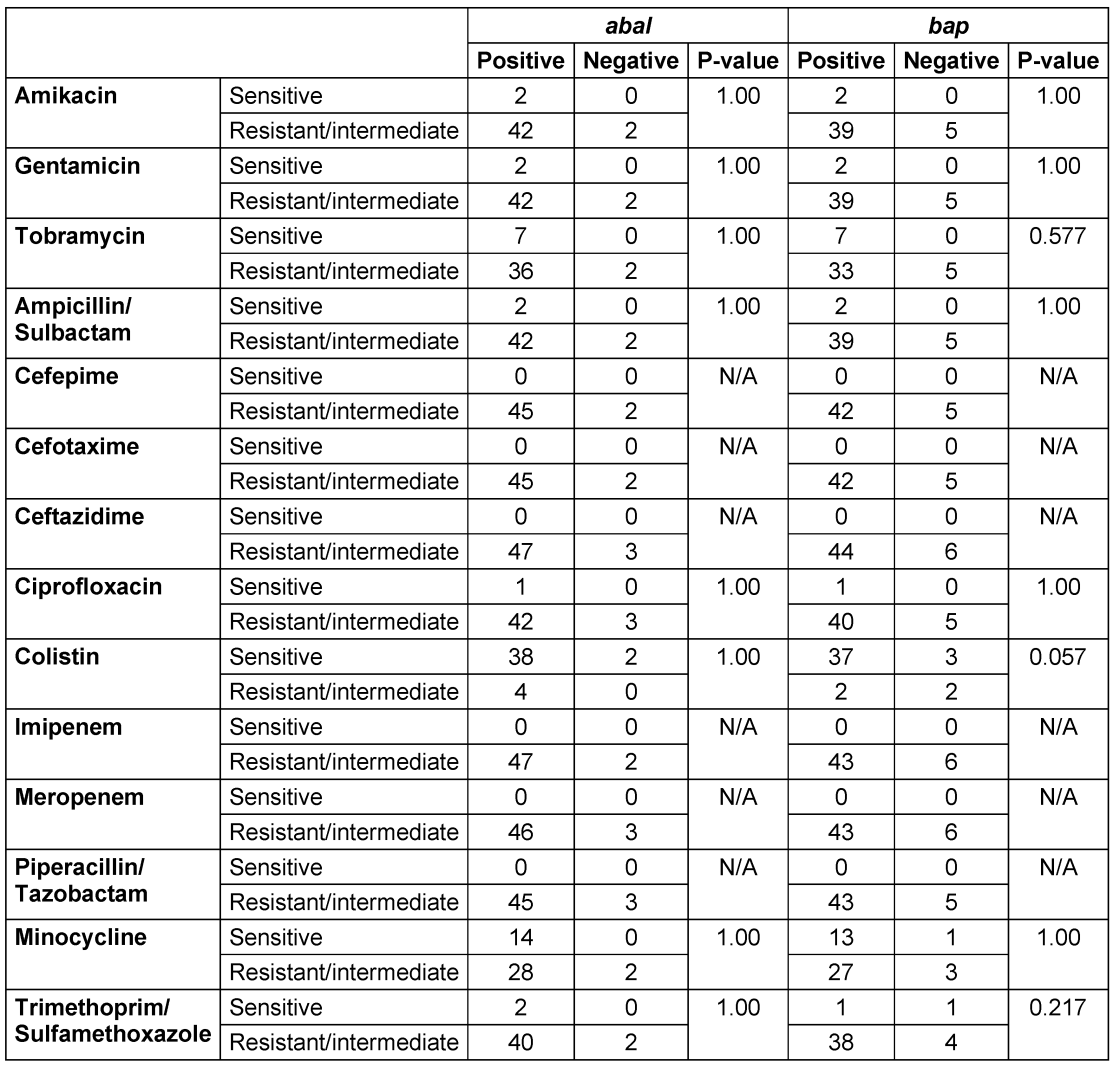
The relationship between the frequency of the virulent genes and the resistance to different antibiotics among the total 50 MDR samples
